# Associations between estimated and measured carotid-femoral pulse wave velocity in older Black and White adults: the atherosclerosis risk in communities (ARIC) study

**DOI:** 10.20517/jca.2021.22

**Published:** 2022-01-04

**Authors:** Kevin Heffernan, Lee Stoner, Michelle L. Meyer, Adam Keifer, Lauren Bates, Patricia Pagan Lassalle, Erik D. Hanson, Masahiro Horiuchi, Erin D. Michos, Anna Kucharska-Newton, Kunihiro Matsushita, Timothy M. Hughes, Hirofumi Tanaka

**Affiliations:** 1 Department of Exercise Science, Syracuse University, Syracuse, NY 13244, USA.; 2 Department of Exercise and Sport Science, University of North Carolina at Chapel Hill, Chapel Hill, NC 27599, USA.; 3 Department of Emergency Medicine, School of Medicine, University of North Carolina at Chapel Hill, Chapel Hill, NC 27599, USA.; 4 Department of Human Environmental Science, Mount Fuji Research Institute, Yamanashi 403-0005, Japan.; 5 Division of Cardiology, Johns Hopkins University School of Medicine, Baltimore, MD 21205, USA.; 6 Department of Epidemiology, The Gillings School of Global Public Health, University of North Carolina at Chapel Hill, Chapel Hill, NC 27514, USA.; 7 Department of Epidemiology, College of Public Health, University of Kentucky, Lexington, KY 40536, USA.; 8 Department of Epidemiology, Johns Hopkins Bloomberg School of Public Health, Baltimore, MD 21205, USA.; 9 Section of Gerontology and Geriatric Medicine, Department of Internal Medicine, Wake Forest School of Medicine, Winston Salem, NC 27157, USA.; 10 Department of Kinesiology and Health Education, The University of Texas at Austin, Austin, TX 78712, USA.

**Keywords:** Vascular stiffness, measurement, health disparities, pulse wave velocity, blood pressure

## Abstract

**Introduction:**

Aortic stiffness offers important insight into vascular aging and cardiovascular disease (CVD) risk. The referent measure of aortic stiffness is carotid-femoral pulse wave velocity (cfPWV). cfPWV can be estimated (ePWV) from age and mean arterial pressure. Few studies have directly compared the association of ePWV to measured cfPWV, particularly in non-White adults. Moreover, whether ePWV and cfPWV correlate similarly with CVD risk remains unexplored.

**Aim:**

(1) To estimate the strength of the agreement between ePWV and cfPWV in both Black and White older adults; and (2) to compare the associations of ePWV and cfPWV with CVD risk factors and determine whether these associations were consistent across races.

**Methods and Results:**

We evaluated 4478 [75.2 (SD 5.0) years] Black and White older adults in the Atherosclerosis Risk in Communities (ARIC) Study. cfPWV was measured using an automated pulse waveform analyzer. ePWV was derived from an equation based on age and mean arterial pressure. Association and agreement between the two measurements were determined using Pearson’s correlation coefficient (*r*), standard error of estimate (SEE), and Bland-Altman analysis. Associations between traditional risk factors with ePWV and cfPWV were evaluated using linear mixed regression models. We observed weak correlations between ePWV and cfPWV within White adults (*r* = 0.36) and Black adults (*r* = 0.31). The mean bias for Bland-Altman analysis was low at −0.17 m/s (95%CI: −0.25 to −0.09). However, the inspection of the Bland-Altman plots indicated systematic bias (*P* < 0.001), which was consistent across race strata. The SEE, or typical absolute error, was 2.8 m/s suggesting high variability across measures. In models adjusted for sex, prevalent diabetes, the number of prevalent cardiovascular diseases, and medication count, both cfPWV and ePWV were positively associated with heart rate, triglycerides, and fasting glucose, and negatively associated with body mass index (BMI) and smoking status in White adults (*P* < 0.05). cfPWV and ePWV were not associated with heart rate, triglycerides, and fasting glucose in Black adults, while both measures were negatively associated with BMI in Black adults.

**Conclusions:**

Findings suggest a weak association between ePWV and cfPWV in older White and Black adults from ARIC. There were similar weak associations between CVD risk factors with ePWV and cfPWV in White adults with subtle differences in associations in Black adults.

## One sentence summary

Estimated pulse wave velocity is weakly associated with measured carotid-femoral pulse wave velocity in older Black and White adults in ARIC.

## INTRODUCTION

Carotid-femoral pulse wave velocity (cfPWV) is the referent measure of aortic stiffness^[1]^, a pre-clinical measure of vascular aging associated with the development of hypertension, and a predictor of target organ damage^[2,3]^ and future cardiovascular disease (CVD) events^[4,5]^. cfPWV is a fairly well-standardized technique but requires technical proficiency and somewhat expensive equipment that limits its broader application to clinical use. A simpler approach to estimating aortic stiffness would increase the likelihood of integrating this measurement into clinical practice.

A number of technical and procedural modifications, including the use of a thigh cuff, have been attempted to simplify cfPWV^[6]^. Additionally, cfPWV can be estimated (ePWV) using only age and mean arterial pressure (MAP)^[7–11]^. In select cohorts, the relationship between ePWV and cfPWV has been shown to be moderately high (*r* = 0.52–0.67)^[12]^, with emerging studies noting ePWV as a significant predictor of CVD events and all-cause mortality (including following adjustment for age and blood pressure)^[8,11–14]^. The original equation to derive ePWV was developed from European cohort data and included predominantly White participants^[7]^. Therefore, while ePWV is potentially a simple and useful tool for predicting CVD risk, further work is warranted to compare ePWV to cfPWV as a measure of arterial stiffness in non-White populations.

The primary aim of the current study was to determine the strength of the association and level of agreement between ePWV and cfPWV and determine whether association and agreement were consistent across races. The second aim was to compare the strength of associations of ePWV and cfPWV with traditional CVD risk factors and determine whether these associations were consistent across races. These aims were tested using a well-characterized population of older Black and White adults from the Atherosclerosis Risk in Communities (ARIC) Study cohort.

## METHODS

This observational study is reported in accordance with STROBE (STrengthening the Reporting of OBservational studies in Epidemiology) guidelines^[15]^. Participants provided written informed consent, and the study was approved by the Institutional Review Boards at all field centers, coordinating center, and central labs and reading centers. Data availability and detailed policies for requesting ARIC data can be found at https://sites.cscc.unc.edu/aric/pubs-policies-and-forms-pg. Select ARIC data can also be obtained from the NHLBI BioLINCC repository (https://biolincc.nhlbi.nih.gov/home/).

### Participants

The ARIC Study is a population-based, longitudinal study of 15,792 participants aged 45–64 years enrolled between 1987 and 1989 from 4 United States communities (Forsyth County, North Carolina; Jackson, Mississippi; Minneapolis, Minnesota; and Washington County, Maryland). Details of the baseline visit have been previously described^[16]^. The study sample included 5683 participants who had cfPWV measured at visit 5 between 2011 and 2013.

We excluded participants with the following pre-existing conditions due to concerns over the quality of the PWV measures: BMI ≥ 40 kg/m^2^, major arrhythmias (Minnesota codes 8–1-3, 8–3-1, and 8–3-2), Minnesota code 8–1-2 with evidence of biased PWV waveforms, aortic aneurysms, abdominal aorta ≥ 5 cm, history of aortic or peripheral revascularization or aortic graft, aortic stenosis, and moderate or greater aortic regurgitation. Additionally, we excluded participants whose race was other than White or Black (due to small sample size), with missing PWV or vascular risk factor data, as well as those with outlying PWV values, defined as PWV values > 3 standard deviations above or below the mean. Thus, of the original 5683 participants, 1205 were excluded because they had one or more of the exclusion conditions: pre-existing conditions (*n* = 579), missing cfPWV data (*n* = 433), cfPWV values 3 SDs away from the mean (*n* = 33), race other than White or Black (*n* = 13), missing risk factor data (*n* = 135), and missing covariates (*n* = 12).

### Study design

Participants were asked not to consume food or drink, and refrain from tobacco and vigorous physical activity after midnight prior to the clinic visit or 8 h prior to the visit. Visit 5 study examination included interviewer-administered questionnaires to obtain demographic data, medical history and lifestyle information, blood and urine collection, and assessment of vascular risk factors and cardiovascular phenotypes, including PWV.

### Outcome measures

#### Carotid-Femoral pulse wave velocity

Following 5–10 min of supine rest, technicians measured cfPWV following a standardized protocol with the automated pulse waveform analyzer VP-1000 Plus (Omron, Kyoto, Japan)^[17]^. cfPWV was estimated as the distance between two arterial recording sites divided by transit time. The distance from the carotid to the femoral artery was directly measured with a segmometer (Rosscraft, Surrey, Canada) and calculated as the carotid to femoral distance minus the distance between the suprasternal notch and the carotid applanation site. To calculate transit time, arterial waveforms were simultaneously acquired for 30 s by applanation tonometry sensors placed on the left common carotid artery (via neck collar) and left common femoral artery. A minimum of two measurements was taken per participant, and the last two measurements were averaged. The validity and reliability of the automatic device for measuring PWV have been described previously^[18]^. Quality assurance for PWV included central training and recertification, quarterly equipment calibration, and ongoing quality control reviews by one of the authors (Tanaka H) on a stratified random sample of 40 records per month, with feedback provided to technicians. Approximately 78% of records were considered optimal quality, 17% were good quality, 3% were acceptable, and none were poor or unacceptable.

#### Estimated pulse wave velocity

Three seated blood pressure (BP) measurements were obtained after a 5 min rest using an oscillometric automated sphygmomanometer (Omron HEM-907 XL, Omron, Kyoto, Japan), and the average of the last 2 measurements was used. ePWV was calculated using equations published by the Reference Values for Arterial Stiffness Collaboration^[7]^, which use age and MAP and take nonlinearity and interactions into account.


$$\text{ePWV}=\;\left(1\right)\;\mathrm{Version}\;1\;\left(\mathrm{V1}\right)\;\mathrm{7.84}-\mathrm{0.33}\times \mathrm{age}+\mathrm{3.8}\times 10^{-3}\times \mathrm{ag}e^{2}-1.\mathrm{97}\times 10^{-5}\times age^{2}\times MAP+2.5\times 10^{-3}\times \left(age\times MAP\right)-1.9\times 10^{-3}\times MAP\;\left(2\right)\;\mathrm{Version}\;2\;\left(V2\right)\;9.\mathrm{587}-0.\mathrm{402}\times age+4.\mathrm{560}\times 10^{-3}\times age^{2}-2.\mathrm{621}\times 10^{-5}\times age^{2}\times MAP+3.\mathrm{176}\times 10^{-3}\times age\times MAP-1.\mathrm{832}\times 10^{-2}\times MAP$$

Mean arterial pressure was calculated from systolic blood pressure (SBP) and diastolic blood pressure (DBP) as follows: DBP + 0.4 (SBP - DBP), where SBP - DBP is pulse pressure. We chose a form factor of 0.4 for the calculation of MAP in agreement with that used by the Reference Values for Arterial Stiffness Collaboration^[7]^. There is currently no consensus on the optimal formula to estimate MAP from SBP and DBP. A traditional 0.33 form factor may be more appropriate for younger adults with higher SBP amplification, while a form factor 0.4 may be more optimal for those with lower SBP amplification (i.e., older adults)^[19]^. It should be noted that MAP calculated from a form factor of 0.41 has previously been shown to be more closely associated with target organ damage than a traditional form factor of 0.33^[20]^. Equation 1 (V1), as used by Vishram-Nielsen *et al.*^[13]^ in the MOnica Risk, Genetics, Archiving, and Monograph Prospective Cohort Project, utilizes a regression equation derived from a reference population with low-moderate CVD risk. We also explored a second regression equation used by Greve *et al.*^[12]^ that was derived from a cohort with higher CVD risk. This particular equation was shown to predict survival in the Systolic Blood Pressure Intervention Trial (SPRINT) study involving hypertensive patients^[14]^.

### Predictor variables: traditional cvd risk factors

For aim 2, the cross-sectional associations between ePWV and cfPWV with the following traditional risk factors were explored: BMI, current smoking, heart rate, blood glucose, triglycerides, high-density lipoprotein (HDL) cholesterol, and low-density lipoprotein (LDL) cholesterol. Although heart rate is not a traditional CVD risk factor, it has been shown to be an important correlate of cfPWV^[21,22]^. Body weight was measured to the nearest 0.1 kg, and height was recorded to the nearest centimeter. BMI was calculated using height and weight. The history of smoking was self-reported and analyzed as dichotomous (current *vs.* noncurrent). Blood samples were obtained following a standardized venipuncture protocol and shipped weekly to ARIC central laboratories, where assays for total cholesterol, HDL cholesterol, triglycerides, and fasting glucose concentration were performed. Total plasma cholesterol concentrations were determined enzymatically^[23]^ using a Cobas-Bio analyzer with reagents purchased from Boehringer Mannheim Biochemicals, (Indianapolis, IN). Plasma LDL cholesterol, concentration was calculated using the Friedewald equation^[24]^, and HDL concentrations were measured using the method of Warnick *et al*.^[25]^.

### Covariate measurements

Age was calculated from the date of birth. Sex and race were self-reported. Participants were asked to bring all prescription and nonprescription medications taken within 2 weeks. That information was transcribed and categorized using MediSPAN prescription codes and classified into medication categories. Participants also self-reported medication use. Medications used included β-blockers, 03B1-blockers, calcium channel blockers, diuretics, angiotensin-converting enzyme inhibitors, and angiotensin II receptor blockers. Diabetes was defined as fasting glucose ≥ 126 mg/dL, non-fasting glucose ≥ 200 mg/dL, anti-diabetic medication use, or self-reported diagnosis of diabetes. Hypertension was defined as SBP ≥ 140 mmHg or DBP ≥ 90 mmHg, or antihypertensive medication use. Prevalent coronary heart disease and stroke were defined by ARIC cohort surveillance data at Visit 5. Prevalent heart failure was defined as physician-reported heart failure or a hospitalization discharge with an ICD code 428.x.

### Statistical analyses

Statistical analyses were performed using R Statistical Software. The α-level was set a priori for all statistical procedures at 0.05. Cumulative frequency and Q-Q plots were used to compare the distributions of cfPWV and ePWV. Participant characteristics were stratified by race and estimated as means and SDs, or frequencies and percent, where appropriate. Race differences in demographic characteristics were assessed using chi-squared tests for categorical variables and independent *t*-tests for continuous variables.

For aim 1, we used linear regression to test whether race moderates the relationship between ePWV and cfPWV. Nonlinearity was explored by specifying the ePWV quadratic term. Subsequently, the correlation between the two measurements was determined by calculating the Pearson product-moment correlation (*r*) and standard error of estimate (SEE). Although there is no universal criterion, in general, *r* value estimates of < 0.2, 0.2–0.4, 0.4–0.70, 0.70–0.9 and > 0.9 indicate negligible, weak, moderate, strong, and very strong correlation, respectively^[26]^. The SEE represents the average distance that the observed values fall from the regression line, with smaller values indicating that the observations are closer to the fitted line. The SEE was calculated using the equation:

$$SD\times \sqrt{\left(1-{\text{r}}^{2}\right)}$$

whereby *SD* is the standard deviation of the criterion measure and r is the Pearson product-moment correlation between test and criterion devices. The relative standard error was also calculated by expressing SEE relative to the mean of cfPWV. Bland-Altman plots were generated to permit visual analysis of the uniformity of error over the range of participant measurement values^[27]^.

For aim 2, associations between traditional risk factors with ePWV and cfPWV were evaluated using linear mixed regression models with field center specified as a random intercept. Independent variables included BMI, current smoking, heart rate, glucose, HDL cholesterol, LDL cholesterol, and triglycerides. Initially, univariate analysis was conducted, in each traditional risk factor was independently regressed against cfPWV. This analysis was repeated for ePWV. Subsequently, a multivariable analysis was performed, with the traditional risk factors simultaneously regressed against cfPWV. The analysis was repeated for ePWV. These models were then adjusted for sex, prevalent diabetes, number of prevalent cardiovascular diseases (hypertension, coronary heart disease, heart failure, stroke), and medication count. Adjusted variable models were further stratified by race. Assumptions of linearity, collinearity, homoscedasticity, and outliers were assessed for each model. We report β coefficient estimates, their precision (95%CI), and the *R*^2^ values for the models.

## RESULTS

### Participants

Descriptive characteristics are reported in Table 1. Following exclusions, the study sample included 4478 participants between the ages of 66 and 90 years, of which 59% were women and 23% were Black. Black participants had higher mean cfPWV (Δ = 0.9 m/s, 95%CI: 0.7–1.1) and ePWV (0.1 m/s, 95%CI: 0.0–0.2) values compared to Whites, although distributions were similar for each race [Supplementary Figure 1]. Compared to Whites, Black participants had higher (worse) MAP (*P* < 0.001), BMI (*P* < 0.001), heart rate (*P* < 0.001), fasting glucose (*P* < 0.019) and LDL-cholesterol (*P* < 0.001) but had more favorable HDL cholesterol (*P* = 0.006) and triglycerides (*P* < 0.001). Black participants also had a greater proportion of diabetes (*P* < 0.001), hypertension (*P* < 0.001), heart failure (*P* < 0.001) and stroke (*P* < 0.001), and more Black participants used each class of medication (all *P* < 0.001).

### Agreement between ePWV and cfPWV

Nonlinearity was explored by specifying the ePWV quadratic term for the total population, and in analyses, stratified by race [Supplementary Table 1]. The quadratic term was non-significant for the total sample population, and within the White (*P* = 0.380) and Black strata. Thus, linear models were used for subsequent analyses.

**Figure 1.**
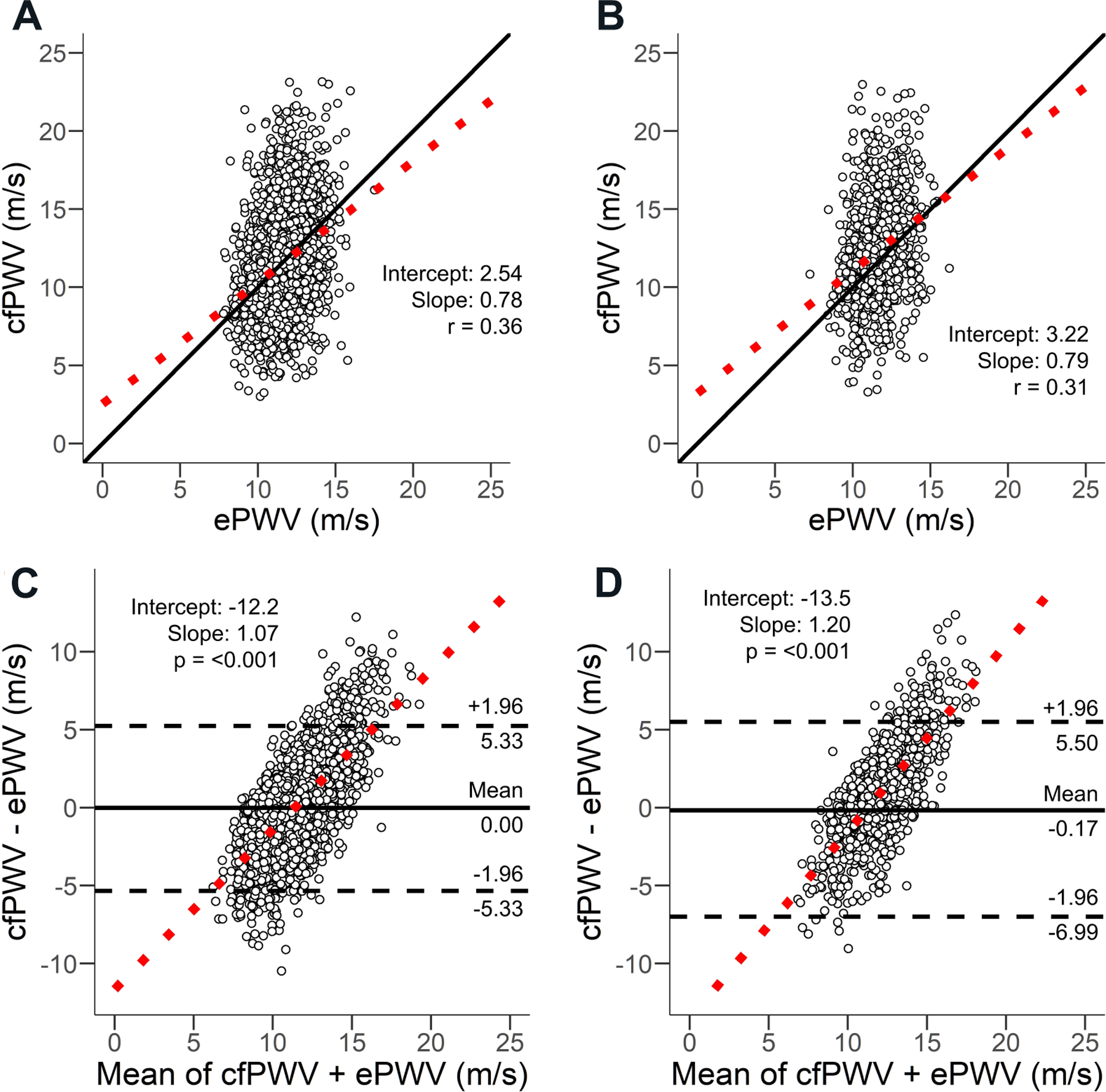
Regression for White adults (A) and Black adults (B). Bland-Altman plots for estimated pulse velocity (ePWV V1) *vs.* carotid-femoral pulse wave velocity (cfPWV) for White adults (C) and Black adults (D). cfPWV: Carotid-femoral pulse wave velocity; ePWV: estimated pulse wave velocity; V1: ePWV equation version 1; V2: ePWV equation version 2.

Correlations between ePWV and cfPWV are reported in Table 2. We observed a weak (*r* = 0.35, 95%CI: 0.32–0.37) correlation between ePWV and cfPWV for the total population, with comparable correlations for White adults (*r* = 0.36, 95%CI: 0.33–0.39) and Black adults (*r* = 0.31, 95%CI: 0.26–0.37). Bland-Altman analysis [Figure 1 and Supplementary Table 2] indicated a mean bias of −0.17 m/s (95%CI: −0.25 to −0.09). However, the small mean bias is misleading; inspection of the regression [Figure 1A and B] and Bland-Altman [Figure 1C and D] plots indicated significant (*P* < 0.001) proportional bias, which was consistent across race strata. The SEE, or typical absolute error, was high at 2.8 m/s suggesting high variability across measures.

Supplementary Table 3 presents the correlation between ePWV using V2 and cfPWV (*r* = 0.35, 95%CI: 0.32–0.37), which was comparable to the correlations between ePWV using V1 and cfPWV, including when stratified by race.

### Correlations between cfPWV and ePWV with traditional cardiovascular disease risk factors

Table 3 presents the associations between traditional cardiovascular disease risk factors and cfPWV and ePWV. In adjusted models (sex, prevalent diabetes, number of prevalent cardiovascular diseases, and medication count), cfPWV was positively associated with heart rate, triglycerides, and fasting glucose, and negatively associated with BMI, HDL cholesterol, and smoking status. ePWV was also positively associated with heart rate and triglycerides, and negatively associated with BMI and smoking status. However, ePWV was not associated with HDL cholesterol or fasting glucose. Neither PWV measure was associated with LDL cholesterol levels in adjusted models. Table 4 presents the associations between traditional cardiovascular disease risk factors with cfPWV and ePWV stratified by race. For Whites, the associations were consistent with those reported for the total population. For Black adults, triglycerides and smoking status were not associated with cfPWV. HDL cholesterol was not associated with ePWV for the total or White populations. HDL showed a positive association with ePWV in Black adults. Similarly, across subgroups, cfPWV and ePWV measures were inversely associated with BMI and smoking status, which is unexpected. Table 5 displays the comparison of ePWV to cfPWV using ARTERY Society Guidelines^[28]^. In general, accuracy across measures was considered acceptable-excellent (mean difference between measures < 1.0 m/s) in approximately 30% of ARIC participants.

Supplementary Table 4 presents the associations between traditional cardiovascular disease risk factors with ePWV V2 for the total group and stratified by race. The findings are consistent with those for ePWV V1. Supplementary Table 5 presents the association of age, MAP, and age-MAP interaction term with cfPWV for the total group and stratified by race.

## DISCUSSION

This study investigated the association between ePWV and cfPWV in White and Black older adults from ARIC. Our primary finding is that ePWV and cfPWV are weakly correlated in older adults, and these weak associations are similar in older Black and White adults.

### Comparison to literature

To generate the equations for ePWV, the Reference Values for Arterial Stiffness Collaboration culled data from 16,867 participants between the ages of 15–97 years (mean age 50 ± 17, equal proportions male and female, 24% smokers) across 13 different clinical centers spanning 8 different European countries. Participants with type 2 diabetes mellitus, overt CVD and adults being treated for hypertension or dyslipidemia were then excluded resulting in a final study population of 11,092 adults^[7]^. Greve *et al.*^[12]^ compared ePWV and cfPWV in both the Danish MONItoring of trends and determinants in CArdiovascular disease (MONICA) 10 cohort (*n* = ~2300 individuals from Copenhagen) and the Paris cohort (*n* = ~1000 adults with essential hypertension), reporting a moderate correlation between the two measures (*r* range: 0.52 to 0.67). Hametner *et al.*^[29]^ compared ePWV to invasively measured aortic PWV measured during cardiac catheterization and noted a similar correlation between ePWV and aortic PWV (*r* = 0.67). Association between ePWV and cfPWV was assessed in a subset from this study, and correlations were reported to be slightly lower (*r* = 0.54)^[29]^. Stamatelopoulos *et al.*^[30]^ recently compared ePWV to cfPWV in 934 adults from the Athens Vascular Registry (mean age 60 years) and noted a correlation of *r* = 0.64. Correlations seen herein in ARIC were appreciably lower than previously reported. The original equation to derive ePWV was developed from European cohort data, and researchers stated that subjects other than Caucasians were a small minority in this cohort^[7]^. Therefore, we had originally hypothesized that racial and ethnic variation in arterial stiffness might impact the correlation between ePWV and cfPWV. Black individuals experience hastened rates of vascular aging^[31,32]^. For a given level of BP, Black individuals have stiffer central arteries at every age^[33]^. However, when exploring associations across race in the ARIC cohort, similar weak associations were noted in White and Black adults, suggesting a general lower association between measures irrespective of racial variation. Thus, racial variation may not be a reason for discrepancies in the association between ePWV and cfPWV when comparing findings herein to previous findings^[12]^.

As the initial equations generated by the Reference Values for Arterial Stiffness Collaboration excluded participants with overt CVD and adults being treated for hypertension or dyslipidemia, a potential reason for the weak association between ePWV and cfPWV reported herein may be due to the CVD risk status of participants. As can be seen from our data, nearly 30% of participants in ARIC were diabetic, over 70% were hypertensive, and an additional 20% had a history of coronary artery disease, heart failure, or stroke. Hypertension and diabetes both accelerate arterial stiffening with aging^[34]^. Moreover, medications to treat hypertension, diabetes, and dyslipidemia may all have variable effects on blood pressure and its relationship to arterial stiffness. For example, antihypertensive therapy can lower brachial BP but have negligible effects on aortic stiffness^[35]^. Thus, the relationship between blood pressure and cfPWV may be different across CVD status and subsequent medical management, affecting the association between ePWV and cfPWV. The Reference Values for Arterial Stiffness Collaboration study population was also significantly younger than ARIC participants (mean age 55 years *vs.* 75 years). Thus, discrepancies between ePWV and cfPWV noted herein are likely related to the older age, and higher CVD risk burden of the ARIC cohort compared to other populations studied.

Other reasons for discrepancy across ePWV and cfPWV may be methodological. The method used by ARIC to estimate path length for cfPWV (i.e., carotid-sternal notch distance subtracted from the carotid to femoral distance) differed from the method used by The Reference Values for Arterial Stiffness Collaboration to standardize cfPWV across study sites (i.e., 80% of the direct carotid-femoral path length)^[7]^. Differences between path length measurements can result in differences in PWV values by upwards of 30%^[36]^. Additionally, different methods used to identify the foot of the pressure waveform (i.e., intersecting tangents *vs.* maximal upstroke) may result in PWV differences of 5%−15%^[7]^. Finally, blood pressure and measures of PWV were not performed simultaneously or in the same position (seated *vs.* supine). BP is higher in the seated position, and given the nature of the equations used for ePWV, a difference in MAP of 3–5 mmHg in a 75-year-old can result in a 0.3–0.4 m/s difference in estimated PWV.

This study further examined the association of traditional CVD risk factors with both measures of PWV. We noted heterogeneity in associations between ePWV and cfPWV with CVD risk factors in analyses stratified by race. In general, arterial stiffness is thought to capture the process of arteriosclerosis, an outside-in process related to structural components of the vessel wall^[37]^. Though sharing pathways in common with atherosclerosis (an inside-out process related to lipid accumulation within the vessel wall), arteriosclerosis has distinct pathophysiology^[37]^. As such, traditional atherosclerotic CVD risk factors (with the exception of age and blood pressure) do not always strongly associate with arterial stiffness^[38]^. Thus, similar to cfPWV, ePWV did not correlate well with many traditional atherosclerotic CVD risk factors. In models adjusted for sex, prevalent diabetes, the number of prevalent cardiovascular diseases, and medication count, both cfPWV and ePWV were positively associated with heart rate and triglycerides and negatively associated with BMI and smoking status. Elevated triglycerides may hasten vascular stiffening via detrimental effects on inflammation and endothelial function^[39]^. Heart rate is known to correlate with arterial stiffness^[40]^. Increased heart rate may expose the vessel wall to more cyclic stress and, when combined with higher blood pressure, may hasten fatigue failure and elastin fracture. The finding of a negative association between BMI and PWV across races in our study, although paradoxical, is consistent with previous findings^[41,42]^. In addition to the explanations previously offered in the literature (e.g., the effect of obesity on stroke volume or reverse epidemiology)^[41]^, it is possible that a strong association between path length and height results in collinearity between cfPWV and BMI, altering the directionality of the association. There were also paradoxical negative associations between current smoking status and PWV measures. More research will be needed to explore this smokers paradox.

### Implications and additional considerations

Despite the weak associations between ePWV and cfPWV noted herein, ePWV may still hold promise as a measure of vascular aging and CVD risk. ePWV has been shown to predict cardiovascular and cerebrovascular events and all-cause mortality independent of traditional CVD risk factors (including the following adjustment for age and BP)^[8–14,29,43–45]^. Recently, ePWV was shown to improve risk stratification for all-cause mortality in hospitalized patients with COVID-19 beyond traditional risk factors and risk scores^[30]^. Additionally, reduction of ePWV with antihypertensive treatment in SPRINT predicted survival independent of effects on BP^[14]^. ePWV has been shown to improve the C-index or net-reclassification index when added to conventional CVD risk scores and improves the area under the receiver-operator characteristic curve beyond traditional CVD risk scores, although this is not a universal finding^[11,13,46]^. Interestingly, Greve *et al*.^[12]^ initially demonstrated that ePWV retained independent predictive value when added to models with cfPWV and even improved prediction of CVD events beyond cfPWV. Thus, ePWV may be capturing interactions between age and MAP that reflect other aspects of vascular aging that are only partially captured by cfPWV itself^[47]^.

According to our results [Supplementary Table 5], the correlation between ePWV and cfPWV is similar to that of age + MAP and cfPWV. It is not uncommon for CVD risk factor interactions to predict outcomes more strongly than their constituent components. For example, the interaction of heart rate and systolic blood pressure (i.e., the double product) predicts cardiovascular outcomes and mortality more strongly than heart rate or systolic blood pressure alone and improves prediction when added to heart rate or systolic blood pressure^[48,49]^. As cfPWV is an estimate of aortic stiffness itself that can be influenced by factors other than age and BP (i.e., tortuosity affecting path length), future studies that estimate PWV from cardiac magnetic resonance imaging-based measures of aortic stiffness may also prove valuable. Indeed, ePWV has been proposed as a potentially useful screening tool and “gatekeeper” to help inform additional testing of aortic stiffness with magnetic resonance imaging^[50]^.

### Limitations and strengths

The strengths and limitations of this study need to be addressed to best contextualize the findings. This was a cross-sectional study (non-experimental study design), and our population consisted of older adults with a relatively high CVD risk factor burden, limiting the generalizability of our findings to other populations. While ARIC participants were originally enrolled between 1987 and 1989, cfPWV measures were performed more recently during visit 5 (2011–2013). Thus, our participant population herein is not representative of the general ARIC study cohort at baseline, owing to participant mortality.

Survival bias should be considered when interpreting findings. We excluded participants with BMI > 40 kg/m^2^ and cardiac arrhythmias owing to effects of obesity on pressure waveform quality and concerns with arrhythmias in calculating cfPWV. Therefore, our findings extend to older adults that are not Class 3 obese or have cardiac electrophysiological abnormalities. Additionally, since Black participants in the ARIC cohort predominantly reside in Jackson, MS, the observed associations may not generalize to Black Americans as a demographic group. The study population may be biased through the inclusion of participants who have survived from baseline (1987–1989) to the time of the Visit 5 examination (2011–2013) and are relatively healthier as compared to those who did not participate in the visit. As with any observational study, we cannot rule out the possibility of residual confounding - although we did include several important confounders in our models. A major strength of this study is that it is the largest study to directly compare ePWV and cfPWV assessments in both White and non-White individuals. Finally, it should be underscored that we are not proposing ePWV as a surrogate for or replacement of cfPWV, but rather as a tool to increase awareness on the importance of vascular aging as it relates to CVD risk and as a possible screening aid to help inform additional vascular aging testing with cfPWV or an equivalent method as needed.

### Conclusions

In conclusion, ePWV is weakly associated with cfPWV in older White and Black adults from the ARIC study.

## Supplementary Material

Supplementary Materials

## Figures and Tables

**Table 1.** Descriptive characteristics of ARIC visit 5 participants, stratified by sex

Continuous variables	Total (*n* = 4478)		White (*n* = 3468)		Black (*n* = 1010)		*P*	*d*
	Mean (SD)		Mean (SD)		Mean (SD)			
Age (years)	75.2	(5.03)	75.5	(5.04)	74.5	(4.94)	< 0.001	0.19
cfPWV (m/s)	11.6	(3.02)	11.4	(2.90)	12.3	(3.34)	< 0.001	0.29
ePWV V1 (m/s)	11.4	(1.33)	11.4	(1.33)	11.5	(1.32)	0.004	0.10
ePWV V2 (m/s)	11.8	(1.37)	11.8	(1.38)	11.9	(1.36)	0.021	0.08
Systolic blood pressure (mmHg)	130	(17.6)	129	(17.3)	134	(18.1)	< 0.001	0.27
Diastolic blood pressure (mmHg)	66.1	(10.4)	65.1	(10.2)	69.4	(10.4)	< 0.001	0.42
Pulse pressure (mmHg)	63.9	(14.3)	63.8	(14.2)	64.3	(14.7)	0.392	0.03
Mean arterial pressure (mmHg)	91.6	(11.8)	90.6	(11.6)	95.1	(12.0)	< 0.001	0.38
Body mass index (kg/m^2^)	27.8	(4.47)	27.4	(4.31)	29.2	(4.31)	< 0.001	0.39
Heart rate (bpm)	64.7	(10.7)	64.3	(10.5)	66.0	(11.4)	< 0.001	0.16
Fasting glucose (mmol/L)	6.21	(1.45)	6.18	(1.40)	6.30	(1.62)	0.019	0.08
LDL Cholesterol (mmol/L)	2.74	(0.89)	2.71	(0.89)	2.86	(0.89)	< 0.001	0.16
HDL Cholesterol (mmol/L)	1.37	(0.36)	1.36	(0.37)	1.40	(0.35)	0.006	0.10
Triglycerides (mmol/L)	1.39	(0.63)	1.44	(0.65)	1.20	(0.51)	< 0.001	0.40
**Categorical variables**	**No. (%)**		**No. (%)**		**No. (%)**		***P***	**OR**
**Female**	2661	59.4	1993	57.5	668	66.1	< 0.001	1.45
**Current smoker**	263	5.9	197	5.7	61	6.0	0.31	1.16
**Prevalent diabetes**	1314	29.3	909	26.2	405	40.1	< 0.001	1.88
**Prevalent cardiovascular disease**								
Hypertension	3302	73.7	2336	67.4	866	85.7	< 0.001	2.93
Coronary heart disease	618	13.8	514	14.8	104	10.3	0.654	0.65
Heart failure	473	10.6	300	8.7	173	17.1	< 0.001	2.18
Stroke	129	2.9	77	2.2	52	5.2	< 0.001	2.39
Total count (median, Q1, Q3)	1	1, 1	1	1, 1	1	1, 2		
**Cholesterol lowering medications**								
Primary	2466	55.1	1943	56.0	523	51.8	0.023	0.85
Secondary	2338	52.2	1711	49.3	623	61.7	< 0.001	1.71
**Hypertensive medications**								
β-Blocker	1232	27.5	1017	29.3	215	21.3	< 0.001	0.67
α-Blocker	143	3.2	87	2.51	56	5.5	< 0.001	2.35
Diuretic	1678	37.5	1109	32.0	569	56.3	< 0.001	2.96
ACE inhibitor	1336	29.8	986	28.4	350	34.7	< 0.001	1.39
ANG II receptor blocker	726	16.2	492	14.2	234	23.2	< 0.001	1.89
Calcium channel blocker	1072	23.9	674	19.4	398	39.4	< 0.001	2.84
Total count (median, Q1, Q3)	1	0, 2	1	0, 2	2	1, 3		

cfPWV: Carotid-femoral pulse wave velocity; ePWV: estimated pulse wave velocity; V1: ePWV equation version 1; V2: ePWV equation version 2; LDL: low-density lipoprotein; HDL: high-density lipoprotein; ACE: angiotensin converting enzyme; ANG: angiotensin.

**Table 2.** Comparison of estimated pulse-wave velocity (ePWV V1) and carotid-femoral pulse wave velocity (cfPWV), stratified by race

	*n*	cfPWV		ePWV		*r*		SEE		RSE	
		Mean (SD)		Mean (SD)		(95%CI)		(95%CI)		(95%CI)	
Total	4478	11.6	(3.0)	11.4	(1.3)	0.35	(0.32–0.37)	2.8	(2.8–2.9)	24.4	(24.2–24.6)
White	2489	11.4	(2.9)	11.4	(1.3)	0.36	(0.33–0.39)	2.7	(2.7–2.7)	23.8	(23.5–24.0)
Black	1644	12.3	(3.3)	11.5	(1.3)	0.31	(0.26–0.37)	3.2	(3.1–3.2)	25.8	(25.3–26.3)

CI: Confidence interval; SEE: standard error of estimate; *r*: Pearson’s correlation coefficient; RSE: relative standard error.

**Table 3.** Multivariable associations between estimated pulse-wave velocity (ePWV V1) and carotid-femoral pulse wave velocity (cfPWV) with traditional vascular risk factors

	Unadjusted (*n* = 4478)				Adjusted (*n* = 4478)			
	β	SE	Std. β	*P*	β	SE	Std. β	*P*
**cfPWV**		***R*** ^**2**^	**0.11**			***R*** ^**2**^	**0.11**	
Body mass index (kg/m^2^)	−0.082	0.011	−0.134	< 0.001	−0.088	0.011	−0.130	< 0.001
								
Heart rate (bpm)	0.053	0.004	0.171	< 0.001	0.058	0.004	0.207	< 0.001
HDL (mmol/L)	−0.643	0.138	−0.045	< 0.001	−0.300	0.149	−0.036	0.044
LDL (mmol/L)	−0.117	0.052	−0.003	0.023	−0.002	0.053	0.000	0.974
Triglycerides (mmol/L)	0.314	0.078	0.041	< 0.001	0.337	0.078	0.070	< 0.001
Fasting glucose (mmol/L)	0.222	0.032	0.096	< 0.001	0.178	0.032	0.086	< 0.001
Smoker status (current *vs.* noncurrent)	−0.362	0.184	−0.015	0.050	−0.392	0.185	−0.030	0.034
**ePWV**		***R*** ^**2**^	**0.02**			***R*** ^**2**^	**0.06**	
Body mass index (kg/m^2^)	−0.025	0.005	−0.084	< 0.001	−0.024	0.005	−0.080	< 0.001
Heart rate (bpm)	0.004	0.002	0.035	0.019	0.005	0.002	0.042	0.005
HDL (mmol/L)	0.016	0.063	0.004	0.800	0.104	0.067	0.028	0.124
LDL (mmol/L)	−0.008	0.024	−0.005	0.745	0.045	0.024	0.030	0.056
Triglycerides (mmol/L)	0.090	0.035	0.042	0.012	0.070	0.035	0.033	0.047
Fasting glucose (mmol/L)	−0.016	0.014	−0.018	0.258	−0.019	0.014	−0.021	0.189
Smoker status (current *vs.* noncurrent)	−0.529	0.084	−0.093	< 0.001	−0.535	0.084	−0.094	< 0.001

Adjustments: sex; prevalent cardiovascular diseases (hypertension, coronary heart disease, stroke, heart failure); medications (β-blockers, α-blockers, calcium channel, blockers, diuretics). Std. β: Standardized beta; SE: standard error; HDL: high-density lipoprotein; LDL: low-density lipoprotein.

**Table 4.** Multivariable associations between estimated pulse-wave velocity (ePWV V1) and carotid-femoral pulse wave velocity (cfPWV) with traditional vascular risk factors, stratified by race

	*n* = 3468				*n* = 1010			
	β	SE	Std. β	*P*	β	SE	Std. β	*P*
**cfPWV**		***R*** ^**2**^	**0.11**			***R*** ^**2**^	**0.13**	
Body mass index (kg/m^2^)	−0.085	0.012	−0.126	< 0.001	−0.109	0.024	−0.153	< 0.001
Heart rate (bpm)	0.062	0.005	0.223	< 0.001	0.052	0.009	0.178	< 0.001
HDL (mmol/L)	−0.294	0.164	−0.037	0.074	−0.357	0.342	−0.038	0.297
LDL (mmol/L)	−0.012	0.058	−0.004	0.839	0.046	0.122	0.012	0.705
Triglycerides (mmol/L)	0.364	0.083	0.082	< 0.001	0.239	0.224	0.037	0.287
Fasting glucose (mmol/L)	0.181	0.036	0.088	< 0.001	0.163	0.068	0.079	0.016
Smoker status (current *vs.* noncurrent)	−0.401	0.204	−0.032	0.049	−0.388	0.427	−0.028	0.364
**ePWV**		***R*** ^**2**^	**0.07**			***R*** ^**2**^	**0.06**	
Body mass index (kg/m^2^)	−0.028	0.006	−0.090	< 0.001	−0.022	0.010	−0.077	0.025
Heart rate (bpm)	0.007	0.002	0.055	0.001	0.001	0.004	0.010	0.760
HDL (mmol/L)	0.036	0.077	0.010	0.637	0.295	0.137	0.079	0.032
LDL (mmol/L)	0.046	0.027	0.031	0.092	0.056	0.049	0.037	0.259
Triglycerides (mmol/L)	0.105	0.039	0.051	0.007	−0.078	0.090	−0.030	0.384
Fasting glucose (mmol/L)	−0.021	0.017	−0.023	0.205	−0.011	0.027	−0.013	0.691
Smoker status (current *vs.* noncurrent)	−0.530	0.096	−0.092	< 0.001	−0.519	0.172	−0.096	0.003

Adjustments: sex; prevalent cardiovascular diseases (hypertension, coronary heart disease, stroke, heart failure); medications (β-blockers, α-blockers, calcium channel, blockers, diuretics). Std. β: Standardized beta; SE: standard error; HDL: high-density lipoprotein; LDL: low-density lipoprotein.

**Table 5.** Classification of ePWV V1 based on mean differences with cfPWV using ARTERY Society recommendations for comparing the accuracy of devices that measure cfPWV

Proportion		Mean difference	Accuracy

White *n* (%)	Black *n* (%)	cfPWV *vs.* ePWV	Classification
580 (17)	138 (16)	< 0.5 m/s	Excellent
515 (15)	157 (15)	0.5–1.0 m/s	Acceptable
2373 (71)	715 (66)	> 1.0 m/s	Poor

ePWV: Estimated pulse wave velocity; cfPWV: carotid-femoral pulse wave velocity.
