# Supplementary material for: Associations between estimated and measured carotid-femoral pulse wave velocity in older Black and White adults: the atherosclerosis risk in communities (ARIC) study
Source: J Cardiovasc Aging. Author manuscript; Available in PMC 2023 Feb 16. (PMC9934460; doi:10.20517/jca.2021.22)
Supplement: Supplementary Materials [file NIHMS1864408-supplement-Supplementary_Materials.pdf]

SUPPLEMENT

TABLE S1. Linear and non-linear regression estimates for the association between estimated pulse-wave velocity (ePWV V1) and carotid-femoral pulse wave velocity (cfPWV), stratified by race.

	**Total n = 4,478**				**White n= 3,468**				**Black n= 1,010**			
	**β**	**LCI**	**UCI**	***P***	**β**	**LCI**	**UCI**	***P***	**β**	**LCI**	**UCI**	***P***
**Linear**	*r* = 0.35 (95%CI: 0.32-0.37)				*r* = 0.36 (95%CI: 0.33-0.39)				*r* = 0.35 (95%CI: 0.26-0.37)			
Intercept	2.57	1.85	3.29		2.54	1.76	3.31		3.22	1.51	4.94	
ePWV	0.79	0.73	0.85	<0.001	0.78	0.71	0.84	<0.001	0.79	0.64	0.93	<0.001
**Non-Linear**	*r* = 0.35 (95%CI: 0.32-0.37)				*r* = 0.36 (95%CI: 0.33-0.39)				*r* = 0.35 (95%CI: 0.26-0.37)			
Intercept	-0.90	-5.64	3.85		0.30	-4.75	5.36		-4.22	-16.33	7.90	
ePWV	1.39	0.57	2.21	<0.001	1.17	0.29	2.04	0.009	2.07	-0.01	4.14	0.051
ePWV*ePWV	-0.03	-0.06	0.01	0.15	-0.02	-0.05	0.02	0.380	-0.05	-0.14	0.03	0.224

Abbreviations: β, beta coefficient; LCI, lower 95% confidence interval; UCI, upper 95% confidence interval

TABLE S2. Bland-Altman estimates for estimated pulse velocity (ePWV V1) versus carotid-femoral pulse wave velocity (cfPWV), stratified by race.

		Bias			Lower LoA			Upper LoA			Mean
	n =	β	LCI	UCI	β	LCI	UCI	β	LCI	UCI	Diff.
Total	4,478	-0.17	-0.25	-0.09	-5.75	-5.90	-5.61	5.41	5.27	5.56	-1.19
White	3,468	0.00	-0.09	0.09	-5.33	-5.49	-5.18	5.33	5.18	5.48	-2.45
Black	1,010	-0.75	-0.94	-0.55	-6.99	-7.33	-6.66	5.50	5.16	5.83	3.13

Abbreviations: β, beta coefficient; LCI, lower 95% confidence interval; UCI, upper 95% confidence interval

TABLE S3. Linear regression estimates for the association between estimated pulse-wave velocity equation II (ePWV V2) and carotid-femoral pulse wave velocity (cfPWV), stratified by race.

	Total n = 4,478				White n= 3,468				Black n= 1,010			
	β	LCI	UCI	*P*	β	LCI	UCI	*P*	β	LCI	UCI	*P*
	r = 0.35 (95%CI: 0.32-0.37)				*r* = 0.36 (95%CI: 0.33-0.39)				*r* = 0.35 (95%CI: 0.26-0.37)			
Intercept	2.58	1.86	3.30		2.52	1.74	3.29		3.23	3.06	3.40	
ePWV.V2	0.76	0.70	0.82	<0.001	0.75	0.69	0.82	<0.001	0.76	0.61	0.90	<0.001

Abbreviations: β, beta coefficient; LCI, lower 95% confidence interval; UCI, upper 95% confidence interval

TABLE S4. Multivariable associations between estimated pulse-wave velocity version 2 (ePWV V2) and carotid-femoral pulse wave velocity (cfPWV) with traditional vascular risk factors, stratified by race.

	Total				White				Black			
	n = 4,478				n = 3,468				n= 1,010			
	β	SE	Std. β	P	β	SE	Std. β	P	β	SE	Std. β	P
ePWV .V2		R^2 =	0.06			R^2 =	0.07			R^2 =	0.06	
Body Mass Index (kg/m^2)	-0.027	0.005	-0.088	< .001	-0.032	0.006	-0.099	< .001	-0.024	0.010	-0.083	0.016
Heart rate (bpm)	0.005	0.002	0.040	0.008	0.007	0.002	0.053	0.002	0.001	0.004	0.009	0.772
HDL (mmol/l)	0.095	0.069	0.025	0.173	0.027	0.080	0.007	0.739	0.294	0.141	0.077	0.037
LDL (mmol/l)	0.040	0.024	0.026	0.105	0.039	0.028	0.025	0.164	0.050	0.051	0.033	0.320
Triglycerides (mmol/l)	0.064	0.036	0.029	0.077	0.102	0.040	0.048	0.011	-0.085	0.093	-0.032	0.362
Fasting glucose (mmol/l)	-0.020	0.015	-0.021	0.178	-0.024	0.017	-0.024	0.175	-0.010	0.028	-0.012	0.711
Smoker Status (current vs. noncurrent)	-0.566	0.086	-0.096	< .001	-0.564	0.099	-0.094	< .001	-0.546	0.177	-0.098	0.002

Adjustments: sex; prevalent cardiovascular diseases (hypertension, coronary heart disease, stroke, heart failure); medications (β-blockers, α-blockers, calcium channel, blockers, diuretics).

TABLE S5. Multivariable associations between components of estimated pulse-wave velocity (age and MAP) and carotid-femoral pulse wave velocity stratified by race.

	Total n = 4,478				White n= 3,468				Black n= 1,010			
	r	LCI	UCI	*P*	*r*	LCI	UCI	*P*	*r*	LCI	UCI	*P*
**Model 1: Age Only**												
	0.24	0.21	0.27	<.001	0.27	0.24	0.30	<.001	0.20	0.14	0.26	<.001
**Model 2: MAP Only**												
	0.25	0.22	0.28	<.001	0.23	0.20	0.26	<.001	0.24	0.18	0.29	<.001
**Model 3: Age + MAP**												
	0.35	0.32	0.38	<.001	0.36	0.33	0.39	<.001	0.32	0.26	0.37	<.001
**Model 4: Age + MAP + Interaction Term**												
	0.35	0.33	0.38	<.001	0.36	0.33	0.39	<.001	0.32	0.26	0.37	<.001

Abbreviations: MAP, mean arterial pressures; LCI, lower 95% confidence interval; UCI, upper 95% confidence interval

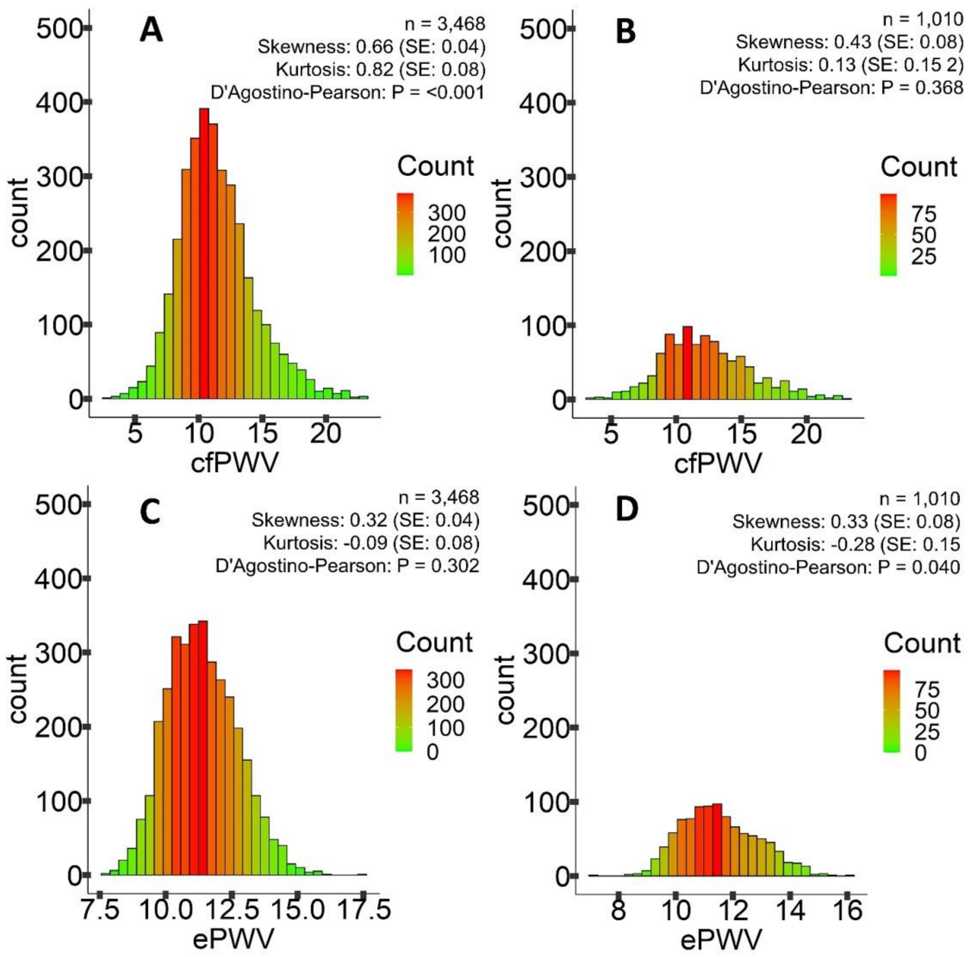
A
n = 3,468
Skewness: 0.66 (SE: 0.04)
Kurtosis: 0.82 (SE: 0.08)
D'Agostino-Pearson: P = <0.001
count
cfPWV
Count
300
200
100
B
n = 1,010
Skewness: 0.43 (SE: 0.08)
Kurtosis: 0.13 (SE: 0.15 2)
D'Agostino-Pearson: P = 0.368
count
cfPWV
Count
75
50
25
C
n = 3,468
Skewness: 0.32 (SE: 0.04)
Kurtosis: -0.09 (SE: 0.08)
D'Agostino-Pearson: P = 0.302
count
ePWV
Count
300
200
100
0
D
n = 1,010
Skewness: 0.33 (SE: 0.08)
Kurtosis: -0.28 (SE: 0.15
D'Agostino-Pearson: P = 0.040
count
ePWV
Count
75
50
25
0

FIGURE S1
